# The effect of high glucose on lipid metabolism in the human placenta

**DOI:** 10.1038/s41598-019-50626-x

**Published:** 2019-10-01

**Authors:** Charlotte H. Hulme, Anna Nicolaou, Sharon A. Murphy, Alexander E. P. Heazell, Jenny E. Myers, Melissa Westwood

**Affiliations:** 1 School of Medical Sciences, Faculty of Biology, Medicine and Health, The University of Manchester, Manchester Academic Health Sciences Centre, Manchester, M13 9PT UK; 2 Maternal and Fetal Health Research Centre, St Mary’s Hospital, Manchester University NHS Foundation Trust, Manchester Academic Health Sciences Centre, Manchester, Manchester, M13 9WL UK; 3 Lydia Becker Institute of Immunology and Inflammation, The University of Manchester, Manchester Academic Health Science Centre, Manchester, M13 9PT UK; 4 School of Heath Sciences, Faculty of Biology, Medicine and Health, The University of Manchester, Manchester Academic Health Science Centre, Manchester, M13 9PT UK

**Keywords:** Endocrine system and metabolic diseases, Reproductive disorders

## Abstract

Diabetes mellitus (DM) during pregnancy can result in fetal overgrowth, likely due to placental dysfunction, which has health consequences for the infant. Here we test our prediction from previous work using a placental cell line that high glucose concentrations affect placental lipid metabolism. Placentas from women with type 1 (n = 13), type 2 (n = 6) or gestational (n = 12) DM, BMI-matched to mothers without DM (n = 18), were analysed for lipase and fatty acid transport proteins and fatty acid and triglyceride content. Explants from uncomplicated pregnancies (n = 6) cultured in physiological or high glucose were similarly analysed. High glucose levels did not alter placental lipase or transporter expression or the profile and abundance of fatty acids, but triglyceride levels were higher (p < 0.05), suggesting reduced β- oxidation. DM did not affect placental protein expression or fatty acid profile. Triglyceride levels of placentas from mothers with pre-existing DM were similar to controls, but higher in obese women with gestational DM. Maternal hyperglycemia may not affect placental fatty acid uptake and transport. However, placental β-oxidation is affected by high glucose and reduced in a subset of women with DM. Abnormal placental lipid metabolism could contribute to increased maternal-fetal lipid transfer and excess fetal growth in some DM pregnancies.

## Introduction

Pregnancies complicated by diabetes mellitus (DM) are associated with adverse perinatal outcomes, most commonly relating to fetal overgrowth (macrosomia). Macrosomic infants can suffer from stillbirth, birth injuries and post-natal metabolic disturbances and are more likely to develop obesity, DM and cardiovascular disease in later life^1^. Maternal hyperglycemia is associated with fetal macrosomia^2^, yet the role of the placenta, the organ responsible for transferring oxygen and nutrients from mother to fetus, in mediating this effect is not well understood. Our previous work using an unbiased systems biology approach to study a placental cell line (BeWo) response to high glucose levels highlighted aberrant trophoblast lipid metabolism and β-fatty acid oxidation^3^.

Maternal plasma free fatty acids are elevated in pregnancies complicated by DM^4^. It is thought that these fatty acids are transferred across the placenta to fetal adipose tissue where they are esterified into triglycerides using glycerol derived from excess maternal glucose, thereby contributing to macrosomia^5^. However, it is possible that maternal glucose may also affect placental lipid metabolism and the transfer of triglycerides from mother to fetus.

In order to enter the placenta, maternal circulating triglycerides carried in lipoproteins must be hydrolysed at the placental membrane by placental lipases (endothelial lipase (EL) or lipoprotein lipase (LPL))^6,7^. While some of the resulting free fatty acids can passively diffuse into the placental trophoblast cells, most require facilitated transport by membrane-bound and intracellular proteins^6^, such as fatty acid translocase (FAT) and fatty acid transporter family proteins (FATP1–6)^6^. Once inside the placenta, fatty acids can be directly transported into the fetal circulation, metabolised via β-oxidation^8^ or stored as triglycerides for subsequent transfer to the fetal circulation. The rate-limiting step for β-oxidation is the conversion of fatty acyl CoA to acylcarnitine in a reaction that is catalysed by carnitine palmityltransferase (CPT1)^8^. This study assessed how high glucose levels affect fatty acid transport and metabolism by placental trophoblast and whether these are disrupted in placentas from women with type 1 and type 2 DM.

## Results

### Effect of high glucose levels on lipid metabolism in placental explants from uncomplicated pregnancies

**Figure 1:**
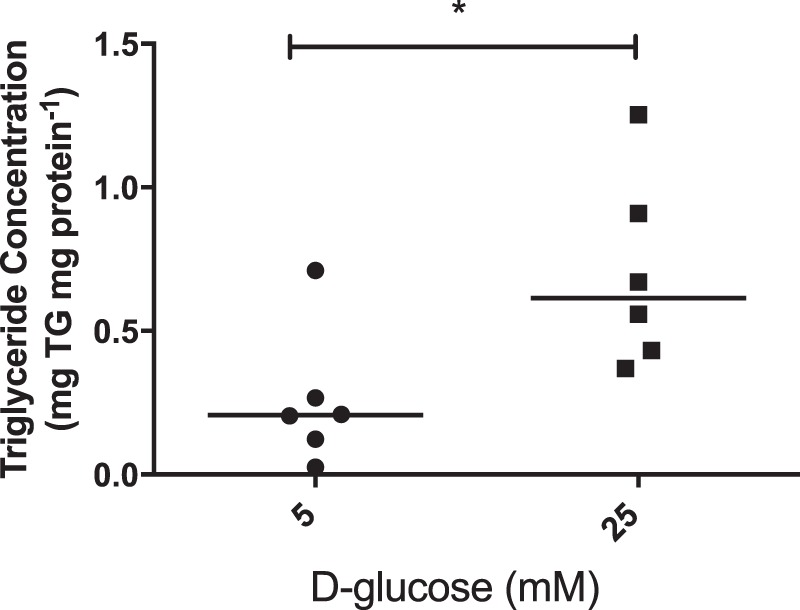
Explants of placenta from uncomplicated pregnancies (n = 6) were cultured for 18 hours in medium containing either 5 mM or 25 mM D-glucose. Explant triglyceride content was measured, in duplicate, using a commercially available kit and concentrations, normalised to protein content, are presented as median and range. *p < 0.05; Mann-Whitney test.

Initially, we sought to confirm our prediction from previous work^3^ – that lipid metabolism is altered in a choriocarcinoma cell-line (BeWo) exposed to high (25 mM) glucose levels - by assessing the effect of such glucose concentrations on the triglyceride content of placental explants isolated from uncomplicated pregnancies at term (Table 1). The data presented in Fig. 1 show that glucose promotes placental triglyceride accumulation; levels were 2.7-fold greater (p < 0.05) in placental explants exposed to high glucose versus explants maintained at physiological glucose concentrations.

**Table 1:** Demographic, obstetric and biophysical data for patient participants with type 1 diabetes (T1DM), type 2 diabetes (T2DM), gestational diabetes and a BMI ≤ 30 (GDM BMI ≤ 30), gestational diabetes and a BMI ≥ 30 (GDM BMI ≥ 30) and BMI matched controls.

	T1DM (n = 13)	T2DM (n = 6)	GDM (BMI < 30) (n = 6)	GDM (BMI ≥ 30) (n = 6)	Control (BMI < 30) (n = 9)	Control (BMI ≥ 30) (n = 9)	*p*
BMI (kg/m^2^)	25 (20–33)	33 (22–41)	26.1 (22–29)	37.9 (32–47)	22 (22–27)	31 (30–32)	ns
Maternal Age (years)	32 (19–43)	38 (26–42)	33 (26–41)	35 (29–41)	34 (29–39)	33 (21–42)	*ns*
Parity	1 (0–5)	2 (0–5)	1 (0–3)	4 (1–9)	1 (0–2)	1 (1–3)	ns
Caucasian (%)	80	83	50	67	77	67	*ns*
Smoker (%)	0	0	0	0	0	0	*ns*
Gestation (week + days)	37 + 1 (36 + 1–39 + 0)	37 + 3 (34 + 0–39 + 0)	38 + 4 (36 + 0–40 + 4)	38 + 4 (38 + 0–40 + 3)	39 + 0 (36 + 5–41 + 4)	39 + 1 (38 + 3–41 + 6)	*ns*
Male (%)	40	83	50	33	33	55	*ns*
Caesarean (%)	87	50	50	83	77	73	*ns*
Birth Weight (g)	3400 (2660–4340)	3209 (2990–3867)	3338 (3035–3720)	4204 (3580–4518)	3080 (2580–3800)	3640 (3180–4200)	*ns*
Birthweight centile^§^	77.5 (43–100)	67.0 (35–95)	62.8 (30–89)	96.7 (94–100)	43 (12–80)	52 (30–88)	a) <0.05
Large (>90^th^) for gestational age (%)	5/13 (42.8)	2/6 (33.3)	0/6 (0)	6/6 (100)	0/9 (0)	0/9 (0)	b) <0.05
HbA1C (3^rd^ trimester) (mmol/mmol)	49 (37–62)	46 (23–72)					

Data are median (range). Abbreviations: BMI, body mass index; ^§^customised birthweight centile^42^ a) - T1 DM compared to controls with a BMI ≤ 30; Kruskal-Wallis test with Dunn’s post-hoc test; b) – Chi-square test.

**Figure 2:**
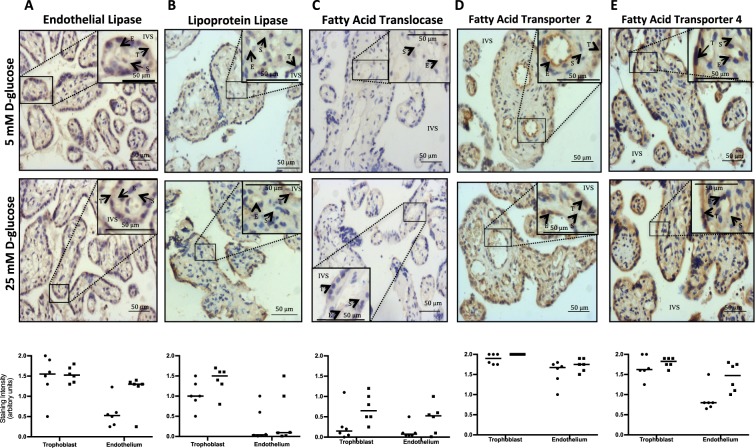
Immunohistochemical analysis of placental tissue from uncomplicated term pregnancies (maternal BMI < 30) cultured in 5 mM (circle) or 25 mM (square) D-glucose for **(A)** endothelial lipase, **(B)** lipoprotein lipase, **(C)** fatty acid translocase, **(D)** fatty acid transporter protein (FATP) 2 and **(E)** FATP4. T – trophoblast, E – endothelium, S - stroma, IVS – intervillous space. Images are representative of data obtained from 6 different placentas. The intensity of staining in the trophoblast and endothelium of explants from each placenta was scored as low (0), medium (1) or high (2) from a mean of ten images; bar represents the median value of 6 placentas. Data were compared using Wilcoxon matched-pairs analysis; no differences in staining for either of the lipases or the fatty acid transporters was observed following explant culture in 5 mM Vs 25 mM D-glucose (p > 0.05).

Next we investigated whether the glucose-mediated increase in placental triglyceride levels was a consequence of altered uptake or transport of fatty acids by determining if glucose affects the expression of EL, LPL, FATP2, FATP4 and FAT, key proteins required for these processes, and/or the level of individual fatty acids accumulated in the placenta^9–12^. Immunohistochemical analyses (shown in Fig. 2) revealed that trophoblast express both EL and LPL in abundance, while endothelium mainly expresses EL (Fig. 2A,B). FAT (Fig. 2C) FATP2 (Fig. 2D) and FATP4 (Fig. 2E) were expressed by all cellular compartments of the placenta, though FATP2 was the most abundant. Treatment of placental explants with high glucose did not alter the localisation or intensity of staining for the lipases or FA transporters (Fig. 2A–E; p > 0.05).

Analysis of the total level of each fatty acid present in placental explants cultured in physiological concentration of glucose (5 mM) (Supplementary Table 1) showed that palmitic acid, stearic acid, oleic acid and arachidonic acid were present in greatest abundance. Neither the profile, nor relative abundance of the fatty acids present in placental explants were affected by exposing tissue to high levels of glucose (Supplementary Table 1; p > 0.05).

**Figure 3:**
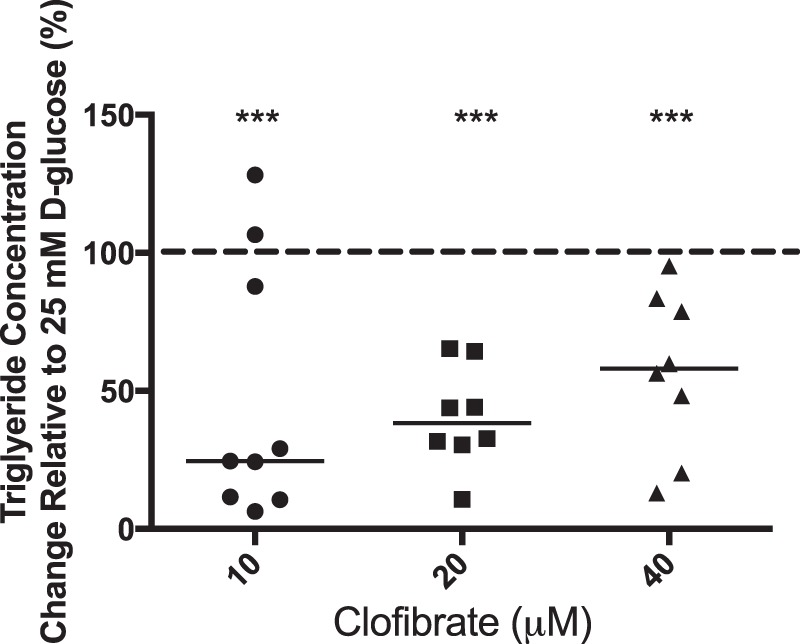
Placental explants from uncomplicated pregnancies (n = 9) were cultured overnight in 25 mM D-glucose and then switched to media containing 25 mM D-glucose in the absence (control) or presence of Clofibrate (10, 20 or 40 μM) for a further 18 hours. Explant triglyceride content was measured, (technical duplicates), using a commercially available colorimetric kit and concentrations were normalised to protein content. Data are presented as % change (median and range) relative to culture in 25 mM D-glucose alone (shown as line at 100%). ***p < 0.001 versus culture in 25 mM D-glucose alone; Wilcoxon-signed rank test.

We therefore explored the possibility that altered placental fatty acid metabolism, specifically their reduced catabolism via β-oxidation, might provide an explanation for the glucose-induced increase in triglyceride levels. The observation that glucose-stimulated accumulation of triglyceride is attenuated in explants co-cultured with all doses of clofibrate (10–40 μM), a PPARα agonist that enhances the expression of CPT1, the rate limiting step for β-oxidation, provides indirect support for this hypothesis (Fig. 3).

### Characterisation of fatty acid uptake and metabolism in placentas from pregnancies complicated by pre-existing maternal DM

**Figure 4:**
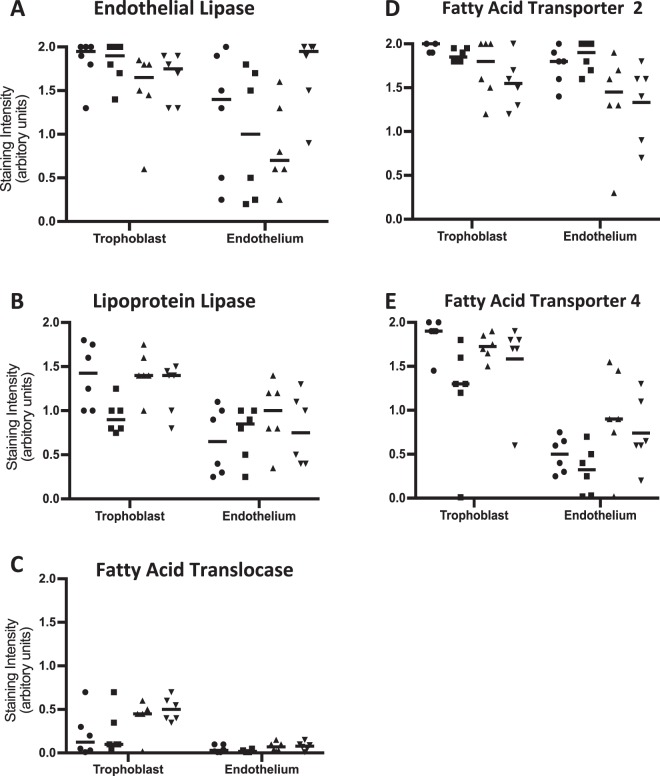
Placental tissue from pregnancies complicated by T1DM (n = 6) or T2DM (n = 6) and uncomplicated term pregnancies (maternal BMI < 30 (n = 6) and >30 (n = 6)) were stained using antibodies that recognise **(A**) endothelial lipase, **(B)** lipoprotein lipase, **(C)** fatty acid translocase, **(D)** fatty acid transporter protein (FATP) 2 or **(E)** FATP4 then analysed by scoring the intensity of staining in the trophoblast and endothelium as low (0), medium (1) or high (2). Data points represent the mean score from ten images, with the median value plotted as a bar. Circle - T1DM; square - T2DM; triangle - control BMI < 30; inverted triangle - control BMI > 30. Data were compared using the Kruskal-Wallis test; no differences in staining for the lipases or fatty acid transporters were observed, in either compartment, between the subject groups (p > 0.05).

Our assessment of the expression of EL, LPL and fatty acid transporters in placentas obtained from women with T1DM or T2DM (Table 1) suggested that both the location and level of each of these proteins are similar to that observed in placentas from BMI-matched uncomplicated pregnancies (p > 0.05; Fig. 4). Similarly, the profile and level of fatty acids found in placental tissue was not affected by the presence of maternal DM (Supplementary Table 2; p > 0.05; Kruskal-Wallis).

**Figure 5:**
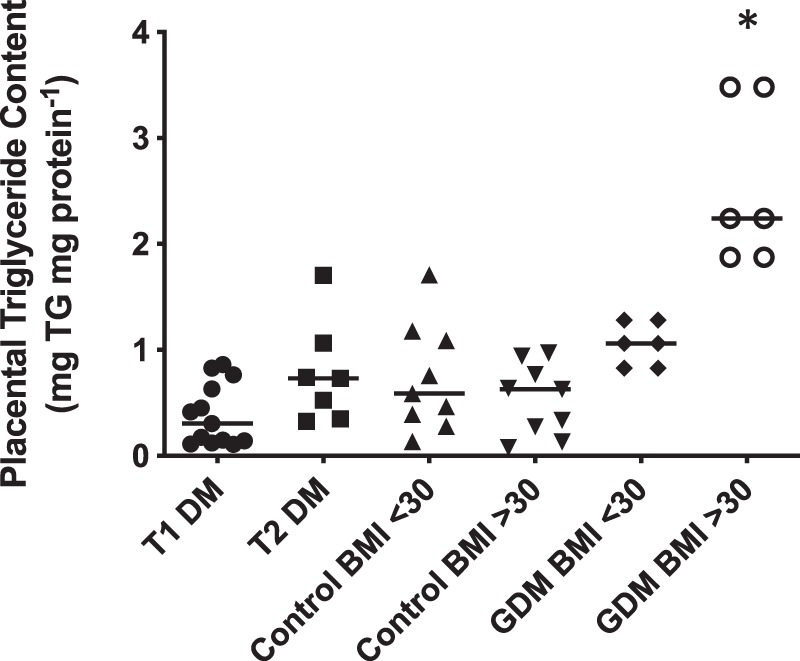
Placental tissue was collected from women with type 1 DM (T1 DM; n = 13), type 2 DM (T2 DM; n = 6), GDM (BMI < 30 (n = 6), BMI > 30 (n = 6)) and BMI matched controls (BMI < 30; n = 9) and (BMI ≥ 30; n = 9). Triglyceride content of each placental homogenate was measured using a commercially available kit and concentrations, normalised to protein content; bar – median. *p < 0.05 Vs control BMI > 30, Kruskal-Wallis with Dunn’s post-hoc test.

Surprisingly, analysis of the triglyceride content of explants from mothers with DM revealed that neither T1DM nor T2DM affected placental triglyceride accumulation when compared to their respective BMI-matched controls (Fig. 5). These data contrast with the observations made in explants exposed to high (25 mM) glucose levels *ex vivo* and therefore, as a control, we also assessed the level of triglycerides in placentas obtained from pregnancies complicated by gestational DM as a previous study reported elevated levels in such tissue^13^. We also found the level of triglycerides in placentas from mothers with gestational diabetes to be significantly higher but only in those with concurrent obesity (Fig. 5).

## Discussion

Our previous systems biology analysis of a trophoblast cell line (BeWo) exposed to high glucose levels predicted alterations in placental lipid metabolism^3^ that could contribute to the enhanced fetal growth often observed in pregnancies complicated by maternal DM. Our current analysis of human placental explants suggests that placental lipid metabolism, but not uptake or transport, is affected by supraphysiological glucose levels; however, this aspect of placental function appears to be maintained in women who have received prolonged hypoglycaemic treatment during pregnancy.

Our immunohistochemical analysis of placental lipase expression revealed that both EL and LPL are expressed by trophoblast. These data suggest that, in contrast to previous hypotheses^7^, EL also has an important role to play in the hydrolysis of maternal lipoproteins as the lysophospholipids generated by EL activity can be further hydrolysed (by EL) to provide a source of fatty acids for the placenta^14^; this suggestion is further supported by reports of an adequate supply of FA to the fetus in women with LPL deficiency^15^, and, importantly, unlike LPL, EL expression by trophoblast is maintained through to term^11^.

We found no evidence of altered lipase expression in placentas exposed, albeit for relatively short time periods, to high glucose *ex vivo*, though our data on *in vivo* exposure (from placentas from women with DM) support this finding. Our data on placental lipase expression corroborate previous reports relating to LPL expression in placentas from women with type 1 DM^16,17^, but others have found higher EL expression in placentas from such pregnancies, particularly in women with poor glycemic control^16^. Therefore, it is possible that satisfactory glycaemic control during pregnancy, which was evident amongst the women in our study, enables maintenance of appropriate EL expression.

The expression of fatty acid transporter proteins FAT, FATP2 and FATP4 in placentas from pregnancies complicated by DM has not been assessed previously, though others have demonstrated increased expression of FATP2 at the basal (fetal-facing) membrane in pregnancies complicated by obesity (defined in that study as BMI > 25)^18^. Our study did not corroborate this finding as the expression of FATP2 in the trophoblast of placentas from women with a BMI ≥ 30 was similar to that observed in tissue from women with a normal BMI, although we did not specifically quantify expression at the basal membrane. In fact, none of the fatty acid transporter proteins demonstrated differential abundance as a consequence of *ex vivo* high glucose or *in vivo* exposure. Although we acknowledge that a limitation of our study is that we did not assess whether the activity of the lipases and transporter proteins, which also influences the availability of fatty acids for metabolism, is altered as a consequence of exposure to altered glucose conditions or maternal DM, our data on protein expression coupled with our finding that the profile and concentrations of fatty acids present in the placenta was unaffected by *ex vivo* exposure to high glucose, or by the presence of maternal DM, suggest that changes solely to the glucose environment are unlikely to alter fatty acid transport into and across the placenta. Rather, any such alterations are likely to be the consequence of composite changes to the metabolic milieu^19^, including hyperinsulinaemia and the altered cytokine/chemokine profile reflective of systemic inflammation^20^ in DM. Nonetheless, it may be beneficial to study the content of the syncytial microvillous (maternal-facing) and basal membranes separately as well as whole tissue homogenates, as others have reported altered levels of oleic acid and linoleic acid in the basal membrane of placentas from other pregnancies complications, including fetal growth restriction^21^. However, our study identified palmitic acid, arachidonic acid, a long chain polyunsaturated fatty acid required for fetal neural development^22^, oleic acid and stearic acid to be the most abundant fatty acids in term placental tissue, which is in keeping with previous reports^21^.

Whilst exposure to aberrant glucose levels appears to have no effect on placental fatty acid uptake and/or transport, our data are indicative of an effect on β-oxidation, as reported by others^13^. Term placental explants cultured in high glucose conditions had higher levels of triglycerides, suggesting that under such conditions, cellular energy is provided by glycolysis rather than lipid oxidation, enabling fatty acid esterification for storage in the form of triglycerides^23^. This putative mechanism is supported by data showing that treatment with the PPARα agonist, clofibrate resulted in attenuation of the glucose-stimulated increase in placental triglyceride levels. PPARα up-regulates CPT1^24^, the rate-limiting enzyme for fatty acid β-oxidation by catalysing the conversion of fatty acyl CoA to acetylcarnitine. Similarly, Martinez *et al*., demonstrated reduced triglyceride levels in explants of placenta from control and diabetic rats following culture with clofibrate^23^. Interestingly, PPARα levels are reduced in placentas from streptozotocin-induced diabetic rats ^25^, however activation of the receptor using the specific ligand, leukotriene B_4_, led to a decrease in placental and fetal weight in such animals ^25^, suggesting that reducing the potential for glucose stimulated storage of fatty acids as triglycerides could reduce the availability of fatty acids for transfer to the fetus, thereby preventing excessive fetal growth. Nonetheless, further studies to directly assess the effect of PPARα on CPT1 expression in placenta, as well as the other mechanisms potentially involved in glucose-mediated regulation of CPT1 activity and β-oxidation, e.g., enhanced acetyl CoA carboxylase production of malonyl CoA, are required to confirm our observations.

Contrary to our initial hypothesis, we did not observe increased triglyceride levels in placental explants from women with either type 1 or type 2 DM. This finding was unexpected, as others have reported elevated levels of triglycerides in placentas from women with gestational DM^13^. We therefore assessed the triglyceride content of such placentas as a control for our assay and also found increased levels, though only in tissue from mothers who had GDM and were also obese (BMI > 30). It is possible that our findings in women with pre-existing DM reflect the fact that these women have intensive monitoring and glucose-lowering treatment throughout their pregnancy with the majority achieving at least satisfactory blood glucose control in the third trimester (only two were admitted and delivered before 37 weeks secondary to concerns regarding glyacemic control). It is also likely that *in vivo*, abnormal glucose metabolism is not the sole cause of aberrant lipid metabolism within the placenta and that maternal insulin levels and dyslipidaemia may also be an important determinant of placental fatty acid metabolism. Previous studies have suggested that the balance between lipogenesis and lipolysis in pregnancies complicated by diabetes may be affected by maternal insulin concentrations^26,27^. Consequently, it is possible that lipid metabolism and triglyceride storage within the placentas of pregnancies complicated by type 1 and 2 diabetes may change following prolonged exposure to an altered maternal metabolic environment associated with prolonged hypoglycaemic treatment (insulin ± metformin).

The increase in triglycerides in women with gestational DM who are also obese suggests that obesity is a potentially important confounding factor present in many women with DM. Critically, the mean BMI in women with gestational DM investigated by Visiedo *et al*.^13^ was 25.9 (n = 8) meaning any effect of obesity would not have been evident. In our study, the women with high BMI and GDM, who all had macrosomic babies, may have had longer (unrecognized) exposure to elevated glucose levels without pharmacological treatment, and/or they may represent a severe phenotype within the GDM population. Furthermore, we acknowledge that the glucose concentration used in our *ex vivo* studies, chosen to reflect previous similar studies^13,28,29^ and our initial work indicating aberrant lipid metabolism in response to exposure to high glucose concentrations^3^, is likely higher than levels typically observed in pregnant women. Further studies to elicit the concentration of glucose needed to drive placental fatty acid metabolism towards storage as triglycerides are required to determine the level of hyperglycemia likely to provoke this effect in women with DM. Future studies should also consider fetal sex as data from pigs^30^ and rabbits^31^ suggest that the transfer and storage of fatty acids is higher in female placentas.

In conclusion, we have confirmed that high glucose leads to increased placental triglyceride levels, indicating decreased placental β-oxidation in a subset of women with DM and obesity, which could lead to an increase in lipid transfer and potentially excessive fetal growth. The effect of high glucose on placental β-oxidation can be attenuated using the PPARα agonist clofibrate. It is possible that targeting CPT1 using PPARα agonists could provide a novel therapeutic intervention to prevent excess placental esterification of fatty acids to triglycerides and attenuate the increased fetal growth observed in pregnancies complicated by maternal DM.

## Methods

### Human placental tissue collection and culture

Human placentas were collected following delivery of a singleton infant between 37 to 42 weeks gestation (Table 1) with informed, written consent in accordance with Research Ethics Committee approval (08/H1010/55 + 5, Manchester UK). As women with type 2 DM are often obese and obesity is associated with altered placental structure and function^32–35^ and altered fetal growth^36,37^, placentas were obtained from two groups of women without DM – those with a BMI < 30 and women that were obese (BMI ≥ 30) – resulting in two groups of ‘control’ placentas. Placentas from two groups of women with GDM were also collected (BMI < 30 and ≥30). Women were diagnosed with GDM following an oral glucose tolerance test performed after 24 weeks gestation or following abnormal home blood glucose monitoring in women with a history of prior GDM (n = 3).

In order to obtain an overall assessment of placental function, a full thickness sample (≈1 cm^3^) was cut from the centre, middle and edge of each placenta (along a ‘line’, chosen at random, radiating from the centre) then the three samples were pooled, washed with sterile, cold phosphate buffered saline and processed for subsequent immunohistochemistry or lipid analyses or dissected into explants (as described previously^38^) for immediate culture.

Explants were cultured in 1:1 DMEM:F12, containing 10% FBS and physiological (5 mM) or high (25 mM) levels of D-glucose for 18 h then fixed for immunohistochemistry analysis or processed for triglyceride analysis. In some experiments, explants cultured in high (25 mM) D-glucose were incubated for a further 18 h in the absence or presence of the PPARα agonist (which can upregulate CPT1 expression^24^), Clofibrate (10–40 μM; Sigma Aldrich), then analysed for triglyceride content. Culture for 18 h was used as this time point had previously been used in similar studies to assess the effects of high glucose concentration on placental explants^13^.

### Immunostaining

Immunohistochemical analysis was performed on 5 µm sections of formalin-fixed wax-embedded villous tissue from the three regions of the placenta (centre, middle and edge) using colorimetric detection as described^39^ and the primary antibodies detailed in Table 2. Ten random images per section were captured and staining in trophoblast and endothelium separately quantified using a semi-quantitative scale (0 – no staining; 1 – some staining and 2 – heavy staining) by two scorers blinded to sample identity. The mean score for each placenta was calculated and tissue from different treatment and patient groups were analysed by Wilcoxon matched-pairs and Kruskal-Wallis with Dunn’s post-hoc tests respectively. p < 0.05 was considered to represent a statistically significant difference. Representative images of data obtained when using non-immune mouse or rabbit IgG as a negative control are presented in Supplementary Fig. 1.

**Table 2:** Antibodies used in immunohistochemical analysis of explants of human placenta obtained at term.

Primary Antibody					Secondary Antibody			
Species	Target	Gene ID	Supplier	Working Concentration	Species	Target	Supplier	Concentration
Mouse	Lipoprotein Lipase	LPL	Millipore (#MABC674)	5 µg/ml	Goat	Mouse IgG	DAKO	7.5 mg/ml
Mouse	Fatty Acid Translocase	FAT	Santa Cruz (sc-21772)^43^	1 µg/ml				
Rabbit	Endothelial Lipase	EL	Pierce (#PA1-16799)	5 µg/ml	Swine	Rabbit IgG	DAKO	4.4 mg/ml
Rabbit	Fatty Acid Transporter 4	FATP4	Santa Cruz (#sc-25670)^44^	1 µg/ml				
Goat	Fatty Acid Transporter 2	FATP2	Santa Cruz (#sc-161311)^45^	0.27 µg/ml	Rabbit	Goat IgG	DAKO	3.9 mg/ml
Mouse	Non-immune IgG		Sigma	At same concentration as primary antibody of interest	Goat	Mouse IgG	DAKO	7.5 mg/ml
Rabbit	Non-immune IgG		Sigma	At same concentration as primary antibody of interest	Swine	Rabbit IgG	DAKO	4.4 mg/ml
Goat	Non-immune IgG		Sigma	At same concentration as primary antibody of interest	Rabbit	Goat IgG	DAKO	3.9 mg/ml

### Analysis of placental fatty acid composition

The profile and relative abundance of individual fatty acids in placental lipid extracts was investigated using gas chromatography as described before^40^; briefly: 100 mg of tissue (made up of tissue from the centre, middle and edge of the placenta) was homogenised in ice-cold chloroform:methanol (2:1 v/v) containing the anti-oxidant butylated hydroxytoluene (BHT) (0.01%) using a blade homogeniser (Ystral; 3 × 15 s). The lipid extracts were trans-esterified using a BF_3_-methanol solution (14%) with C21:0 (Supelco) as internal standard. Fatty acid methyl esters (FAME) were analysed on an Agilent 6850 GC-FID gas chromatograph (Agilent Technologies) with a BPX70 capillary column (0.25 μm film, 60 m × 0.25 mm; SGE Europe Ltd) using helium as the carrier gas and a flame ionization detector. Fatty acids were identified by comparison of retention times to those obtained by analysis of FAME standards (Sulpeco, UK). Data reported as relative percentage weight of total fatty acids.

The effect of glucose on the percentage weight of individual fatty acids was assessed using the Wilcoxon-signed rank test. Differences in the percentage weight of individual fatty acids in placental tissue from T1DM and T2DM compared to their BMI-matched controls were measured using a Kruskal-Wallis test with a Dunn’s post-hoc test. p < 0.05 was considered to be statistically significant.

### Analysis of placental triglyceride concentration

Triglycerides were extracted by homogenising placental tissue (20 mg, containing tissue from the centre, middle and edge of the placenta) using an electronic blade homogeniser (LTF Labortechnick, Germany) in HPLC- grade acetone then rotating samples overnight at 4 °C and centrifuging at 15,000 × g for 15 min. Triglyceride concentrations were measured using a commercial colorimetric assay based upon enzymatic hydrolysis of the triglycerides by lipase to produce glycerol and free fatty acids, following manufacturers’ instructions (Caymann Chemical, Cambridge) and are presented as per mg of protein (determined using the Lowry method^41^). The effect of *ex vivo* glucose exposure on placental triglyceride content was assessed using a Mann-Whitney test whereas the Wilcoxon-signed rank test was used to analyse experiments including clofibrate and the levels of triglyceride in placentas from the different patient groups were compared by Kruskal-Wallis. p < 0.05 was considered to represent a significant difference.

## Supplementary information

Supplementary Tables and Figure
