# Supplementary material for: The effect of high glucose on lipid metabolism in the human placenta
Source: Sci Rep. 2019 Oct 1;9:14114. doi: 10.1038/s41598-019-50626-x (PMC6773712; doi:10.1038/s41598-019-50626-x)
Supplement: Supplementary file 1 — Supplementary Tables and Figure [file 41598_2019_50626_MOESM1_ESM.pdf]

The effect of high glucose on lipid metabolism in the human placenta

Charlotte H. Hulme[1,2], Anna Nicolaou[3,4], Sharon A Murphy[4], Alexander E. P. Heazell[1,2], Jenny E. Myers[1,2], & *Melissa Westwood[1,2]

[1]School of Medical Sciences, Faculty of Biology, Medicine and Health, The University of Manchester, Manchester Academic Health Sciences Centre, Manchester M13 9PT, UK;

[2]Maternal and Fetal Health Research Centre, St Mary's Hospital, Central Manchester University Hospitals NHS Foundation Trust, Manchester Academic Health Sciences Centre, Manchester, Manchester M13 9WL, UK;

[3]Lydia Becker Institute of Immunology and Inflammation, The University of Manchester, Manchester Academic Health Science Centre, Manchester M13 9PT, UK.

[4]School of Heath Sciences, Faculty of Biology, Medicine and Health, The University of Manchester, Manchester Academic Health Science Centre, Manchester M13 9PT, UK.

Fatty acid (shorthand notation)	% Weight of Total Fatty Acid		25 mM Vs 5 mM D-glucose (p-value)
	5 mM D-glucose	25 mM D-glucose	
**Myristic acid** (C14:0)	0.60 ± 0.19	0.51 ±0.11	1.00
**Pentadecanoic acid** (C15:0)	0.41 ± 0.41	0.49 ± 0.75	0.94
**Pentadecenoic acid** (C15:1)	0.71 ± 0.43	0.40 ± 0.29	0.31
**Palmitic acid** (C16:0)	25.86 ± 0.87	26.85 ± 0.62	0.67
**Palmitoleic acid** (C16:1)	0.57 ± 0.33	0.38 ± 0.09	0.39
**Heptadecanoic acid** (C17:0)	0.41 ± 0.05	0.47 ± 0.09	0.39
**Heptadecenoic acid** (C17:1)	0.40 ± 0.07	0.34 ± 0.11	0.82
**Stearic acid** (C18:0)	16.08 ± 2.02	17.54 ± 1.57	0.49
**Elaidic acid** (C18:1n-9t)	0.01 ± 0.07	0.00 ± 0.11	0.93
**Oleic acid** (C18:1n-9c)	10.05 ± 2.61	9.54 ± 1.53	0.82
**Vacceinic Acid** (C18:1n-7)	1.44 ± 0.40	1.82 ± 0.86	0.77
**Linolelaidic acid** (C18:2n-6t)	0.06 ± 0.04	0.05 ± 0.04	0.69
**Linoleic acid** (C18:2n-6c)	7.41 ± 1.07	7.52 ± 1.77	0.70
**γ-linoleic acid** (C18:3n-6)	0.23 ± 0.08	0.32 ± 0.30	1.00
**Linolenic acid** (C18:3n-3)	0.13 ± 0.15	0.08 ± 0.09	0.94
**Arachidic acid** (C20:0)	0.27 ± 0.13	0.30 ± 0.10	0.59
**Eicosenoic acid** (C20:1n-9)	0.30 ± 0.34	0.75 ± 1.11	0.48
**Eicosadienoic acid** (C20:2)	0.40 ± 0.07	0.34 ± 0.16	0.94
**Eicosatrienoic acid** (C20:3n-6)	4.34 ± 1.21	3.42 ± 1.83	0.48
**Arachidonic acid** (C20: 4n-6)	18.72 ± 1.51	14.81 ± 6.78	0.94
**Eicosatrienoic acid** (C20: 3n-3)	1.12 ± 0.57	1.71 ± 2.49	0.63
**Behenic acid** (C22:0)	0.31 ± 0.46	0.49 ± 0.40	0.70
**Erucic acid** (C22:1n-9)	0.19 ± 0.05	0.22 ± 0.14	1.00
**Eicosapentaenoic acid** (C20:5n-3)	0.08 ± 0.04	0.09 ± 0.10	0.26
**Tricosanoic acid** (C23:0)	0.29 ± 0.07	0.20 ± 0.12	0.18
**Docosadienoic acid** (C22:2)	0.06 ± 0.02	0.03 ± 0.04	0.23
**Docosatetraenoic acid** (C22:4n-6)	1.49 ± 0.49	1.41 ± 0.73	1.00
**Lignoceric acid** (C24:0)	2.01 ± 1.07	1.75 ± 1.38	0.94
**Nervonic acid** (C24:1)	1.14 ± 0.15	1.00 ± 0.14	0.94
**Docosapentaenoic acid** (C22:5n-3)	0.93 ± 0.14	0.84 ± 0.13	1.00
**Docosahexaenoic acid** (C22:6n-3)	4.39 ± 0.56	4.06 ± 0.61	0.82

Supplementary Table One. Profile of fatty acids present in explants of placenta from uncomplicated pregnancies (n=6). The mean (SD) amount of each fatty acid as percentage weight of total fatty acids in placental explants cultured in 5 mM or 25 mM D-glucose for 24 h is shown. Data were analysed using the Wilcoxon-matched pairs test; no statistically significant differences were detected.

Fatty acid (shorthand notation)	% Weight of Total Fatty Acid				Kruskal -Wallis (p-value)
	T1 DM	Control (BMI ≤30)	T2 DM	Control (BMI ≥30)	
**Myristic acid** (C14:0)	0.39 ± 0.22	0.50 ± 0.26	0.44 ± 0.10	0.43 ±0.06	0.70
**Pentadecanoic acid** (C15:0)	0.65v±0.70	1.30 ± 1.07	0.80 ± 0.87	0.51 ±0.41	0.61
**Pentadecenoic acid** (C15:1)	0.92 ± 1.26	0.92 ± 0.98	0.48 ± 0.47	0.75 ±0.79	0.93
**Palmitic acid** (C16:0)	25.51 ± 0.70	25.47 ± 0.52	25.39 ± 0.75	23.6 ±4.15	0.83
**Palmitoleic acid** (C16:1)	0.44 ± 0.11	0.57 ± 0.52	0.50 ± 0.14	0.43 ±0.19	0.72
**Heptadecanoic acid** (C17:0)	0.29 ± 0.35	0.35 ± 0.05	0.31 ± 0.07	0.53 ± 0.31	0.62
**Heptadecenoic acid** (C17:1)	0.47 ± 0.10	0.42 ± 0.09	0.42 ± 0.08	0.43 ± 0.17	0.76
**Stearic acid** (C18:0)	13.18 ± 0.57	14.73 ± 2.31	17.50 ± 6.30	15.52 ± 1.06	0.59
**Elaidic acid** (C18:1n-9t)	2.08 ± 3.07	1.91 ± 3.07	2.83 ± 4.35	1.35 ± 2.88	0.89
**Oleic acid** (C18:1n-9c)	6.58 ± 3.68	9.49 ± 7.57	7.29 ± 4.57	7.57 ± 2.99	0.85
**Vacceinic acid**(C18:1n-7)	0.96 ± 0.67	1.18 ± 0.57	1.09 ± 0.84	1.12 ± 0.38	0.87
**Linolelaidic acid** (C18:2n-6t)	0.01± 0.02	0.15± 0.30	0.04 ± 0.03	0.02 ± 0.02	0.31
**Linoleic acid** (C18:2n-6c)	9.54± 1.05	8.31 ± 2.42	9.22 ± 1.81	8.93 ± 1.16	0.58
**γ-linoleic acid** (C18:3n-6)	0.12 ± 0.05	0.24 ± 0.38	0.17 ± 0.18	0.06 ± 0.07	0.70
**Linolenic acid** (C18:3n-3)	0.02 ± 0.03	0.06 ± 0.06	0.08 ± 0.12	0.02 ± 0.02	0.65
**Arachidic acid** (C20:0)	0.27 ± 0.09	0.33 ± 0.03	0.32 ± 0.07	0.25 ± 0.07	0.56
**Eicosenoic acid** (C20:1n-9)	0.17 ± 0.07	0.18 ± 0.15	0.47 ± 0.66	0.14 ± 0.03	0.32
**Eicosadienoic acid** (C20:2)	0.37 ± 0.05	0.37 ± 0.07	0.38 ± 0.05	0.31 ± 0.07	0.76
**Eicosatrienoic acid** (C20:3n-6)	4.11 ± 2.00	5.17 ± 0.94	5.01 ± 0.82	4.68 ± 1.98	0.94
**Arachidonic acid** (C20: 4n-6)	20.59 2.42	19.20 ± 2.84	18.63 ± 5.25	18.43 ± 3.92	0.70
**Eicosatrienoic acid** (C20:3n-3)	2.41 ± 2.72	5.17 ± 0.93	5.01 ± 0.82	4.68 ± 1.98	0.82
**Behenic acid** (C22:0)	0.10 ± 0.06	0.09 ± 0.09	0.21 ± 0.28	0.18 ± 0.27	0.90
**Erucic acid** (C22:1n-9)	0.16 ± 0.06	0.13 ± 0.09	0.19 ± 0.07	0.18 ± 0.07	0.40
**Eicosapentaenoic acid** (C20:5n-3)	0.25 ± 0.34	0.04 ± 0.05	0.10 ± 0.06	0.08 ± 0.09	0.32
**Tricosanoic acid** (C23:0)	0.20 ± 0.06	0.24 ± 0.03	0.24 ± 0.07	0.19 ± 0.12	0.65
**Docosadienoic acid** (C22:2)	0.03 ±0.02	0.06 ±0.08	0.21 ±0.44	0.03 ± 0.03	0.74
**Docosatetraenoic acid** (C22:4n-6)	1.12 ±0.22	1.19 ±0.36	1.32 ± 0.17	1.01 ± 0.59	0.39
**Lignoceric acid** (C24:0)	1.69 ±0.38	1.08 ±0.56	1.52 ± 0.34	1.28 ± 0.10	0.58
**Nervonic acid** (C24:1)	1.14 ±0.13	0.87 ±0.11	1.02 ± 0.22	1.14 ± 0.15	0.53
**Docosapentaenoic acid** (C22:5n-3)	0.82 ±0.23	0.71 ±0.18	0.85 ± 0.15	1.25 ± 1.08	0.58
**Docosahexaenoic acid** (C22:6n-3)	4.60 ±0.88	3.47 ±0.46	3.71 ± 0.71	4.21 ± 1.00	0.62

Supplementary Table Two. Profile of fatty acids present in placentas from women with pregnancies complicated by type 1 diabetes (n=6), type 2 diabetes (n=6) and BMI-matched uncomplicated pregnancies (BMI ≤ 30, n=6; BMI ≥ 30, n=6). The mean (SD) amount of each fatty acid as percentage weight of total fatty acids is shown. Data were analysed using the Kruskal-Wallis test; no statistically significant differences were detected.

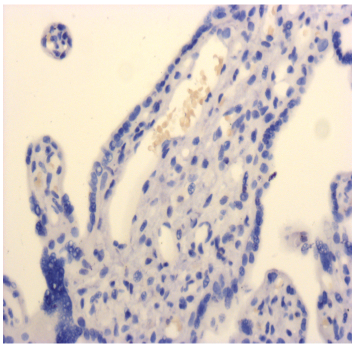

Mouse non-immune IgG (5μg/ml)

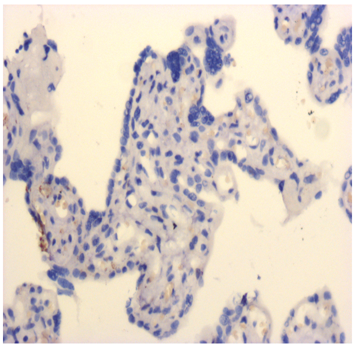

Rabbit non-immune IgG (5μg/ml)

Supplementary Figure One. Immunohistochemical analysis of placental tissue from uncomplicated term pregnancies probed with non-immune IgG from mouse or rabbit in place of protein-specific primary antibody. Images are representative of data obtained from 6 different placentas.
